# Bioactive Properties and Phenolic Profile of Bioaccessible and Bioavailable Fractions of Red Radish Microgreens After In Vitro Digestion

**DOI:** 10.3390/molecules30142976

**Published:** 2025-07-15

**Authors:** Dorota Sosnowska, Małgorzata Zakłos-Szyda, Dominika Kajszczak, Anna Podsędek

**Affiliations:** Institute of Molecular and Industrial Biotechnology, Faculty of Biotechnology and Food Sciences, Lodz University of Technology, Stefanowskiego 2/22, 90-537 Łódź, Poland; dorota.sosnowska@p.lodz.pl (D.S.); malgorzata.zaklos-szyda@p.lodz.pl (M.Z.-S.); dominika.kajszczak@p.lodz.pl (D.K.)

**Keywords:** microgreens, radish, phenolic compounds, bioaccessibility, bioavailability, cytoprotective effect, anti-inflammatory activity, antioxidant activity

## Abstract

The health-promoting activity of radish microgreens after consumption depends on their bioaccessibility and bioavailability. In this study, we compared the composition of phenolic compounds, their cytoprotective and anti-inflammatory activities in cell lines, and antioxidant properties of the undigested radish microgreens with their fractions obtained after simulated in vitro digestion in the stomach, as well as in the small and large intestine. The results have demonstrated higher levels of total phenolics (by 70.35%) and total hydroxycinnamic acids (3.5 times increase), an increase in scavenging efficiency toward ABTS^•+^ and superoxide anion radicals, and an increase in the reduction potential (FRAP method) in the gastric bioaccessible fraction. In contrast, small intestinal digestion negatively affected phenolic content (a reduction of 53.30–75.63%), except for total hydroxycinnamic acids (3-fold increase). Incubation of the non-bioavailable fraction with bacterial enzymes led to further degradation. Undigested microgreens had no negative impact on Caco-2, HT-29, and SH-SY5Y cells’ metabolism at 0.05–2 mg/mL, while all digested samples at 1 mg/mL revealed their cytotoxic potential. All samples used at a non-cytotoxic concentration showed protective activity against H_2_O_2_ and corticosterone-induced oxidative stress generation as well as reduced proinflammatory cytokines production. Overall, radish microgreens may exhibit a broad spectrum of biological activities when consumed.

## 1. Introduction

Microgreens have become more popular worldwide due to their nutritional value and health benefits and can play an important role in dietary diversity. They are defined as plants that are consumed when their first true leaves appear, usually harvested 7 to 21 days after germination, depending on the species. Microgreens can help alleviate food shortages, as they require minimal space, are suitable for hydroponic growing systems, and can be cultivated indoors or in kitchen gardens. Microgreens are known as a fresh, ready-to-eat functional foods, earning the nickname “vegetable confetti”. Many plant species can be cultivated as microgreens, including radish (*Raphanus sativus* L.) [1,2,3]. Radish microgreens have demonstrated various biological activities, including anti-inflammatory [4,5], anticancer [4,5,6,7,8], antidiabetic [5,9,10,11,12], anti-obesity [13], neuroprotective [5,12], antioxidant [5], antihypertension [5], and antimicrobial effects [5,14]. These properties are attributed to the presence of bioactive compounds, such as vitamin C, carotenoids, chlorophylls, glucosinolates, and phenolic compounds. Phenolic compounds are the most abundant heterogeneous group of secondary plant metabolites, characterized by at least one aromatic ring with one or more hydroxyl groups attached, including phenolic acids (hydroxybenzoic and hydroxycinnamic acids), stilbenes, coumarins, flavonoids (flavonols, flavan-3-ols, flavones, flavanones, isoflavones, anthocyanins), lignans, and tannins (both condensed and hydrolysable tannins). Radish microgreens were reported to contain 179 phenolic acids, 174 flavonoids, 88 lignans and coumarins, 15 quinones, and 6 tannins [15]. The potential health benefits of dietary phenolic compounds depend on their absorption and metabolism, which are determined by their structure, including the degree of hydroxylation, glycosylation, and acylation, molecular size, and conjugation with other food matrix components, such as fiber, digestible carbohydrates, proteins, and lipids. It is well established that plant-derived phenolic compounds can undergo diverse digestive transformations catalyzed by digestive enzymes and are further metabolized by the colonic microbiota. In the small intestine, these compounds are often conjugated or modified by β-glucosidases, UDP-glucuronosyltransferases, or catechol-O-methyltransferases, then transported via the portal vein to the liver, where they are further processed by phase I and II enzymes, undergoing methylation, sulfation, or glucuronidation [16,17,18].

The bioavailability of phenolic compounds in the gastrointestinal tract can be investigated using in vitro human digestion models. These digestion–fermentation models simulate the human digestive process, including oral, gastric, and intestinal phases, as well as microbial fermentation in the large intestine [19,20,21]. Several studies have employed such in vitro systems to track the bioaccessibility of selected phenolic compounds from radish microgreens [22,23,24]. For example, Šola et al. [22] observed a decrease in the total phenolics, antioxidant capacity, and α-glucosidase inhibition efficiency, alongside an increase in α-amylase inhibition, in an ethanolic extract of radish microgreens after in vitro gastro-intestinal digestion. Similarly, Tomas et al. [23] reported that in vitro digestion of fresh purple radish microgreens negatively affected their free radical scavenging activity and reduction potential. Moreover, they observed a decrease in the content of total phenolics and total monomeric anthocyanins. They also estimated the in vitro bioaccessibility of total phenolics at 12.1%. In contrast, de la Fuente [24] reported a nearly 68% bioaccessibility of total soluble phenolics in freeze-dried radish microgreens. Additionally, the bioaccessible fraction retained 54.27 and 28.18% of the antioxidant capacity of undigested microgreens as measured by ORAC and ABTS methods, respectively. In a subsequent study, these authors demonstrated a 12.2% reduction in the proliferation of colon cancer Caco-2 cells in response to the digested fraction, with no antiproliferative effects in normal human colon CCD18-Co cells [8]. In summary, the transformation of bioactive phytochemicals during the digestion process can significantly change the properties of radish microgreens determined directly from extracts. Therefore, to obtain results that better reflect in vivo conditions, it is important to assess the biological activity of microgreens after simulated digestion.

Taking this into consideration, the present study employed a static in vitro oral-gastrointestinal digestion model to evaluate the bioaccessibility and bioavailability of phenolic compounds from red radish microgreens, detailing changes in phenolic composition after oral-gastric and small intestine digestion and assessing their antioxidant activity through various assays. In addition, the non-available fraction was incubated with a mixture of bacterial proteases (Pronase E) and a mixture of carbohydrolases (Viscozyme L) to simulate the fermentation process in the large intestine and was analyzed as the resulting bioavailable fraction. To evaluate the cytoprotective and anti-inflammatory effects of both undigested and digested radish microgreens, several cell lines were employed that mimic different aspects of the gastrointestinal system, and gut–immune–brain axis regulation: human intestinal epithelial cells HT-29 and Caco-2, RAW 264.7 macrophages to model the inflammatory responses, and SH-SY5Y neuronal cells. The results obtained may contribute to a deeper understanding of the potential health benefits of *Raphanus sativus* microgreens.

## 2. Results and Discussion

Microgreens offer a year-round fresh food source due to their ease of cultivation and rapid growth cycle. They provide a convenient solution for improving dietary diversity, addressing global malnutrition, and supporting human health. Like other microgreens, those derived from radish seeds are not only nutrient-rich but also contain various classes of bioactive phytochemicals [4,25,26]. The nutritional value and health-promoting activity of radish microgreens after consumption depend on their bioaccessibility and bioavailability. Interactions with food matrix components can affect their solubility and stability during digestion, which ultimately affects their absorption in the gastrointestinal tract [27,28]. In this study, freeze-dried red radish microgreens of the Warta cultivar were analyzed to assess the stability of phenolic compounds, antioxidant capacity, and biological activity across different cell lines during an in vitro digestion process, including a simulation of large intestine conditions.

### 2.1. Composition of Freeze-Dried Radish Microgreens

The content of basic macronutrients, ash, dietary fiber, and antioxidant-related compounds in freeze-dried red radish microgreens is presented in Table 1.

The predominant component in the tested microgreens was dietary fiber (28.37 g/100 g), followed by available carbohydrates (26.17 g/100 g) and total protein (21.05 g/100 g). In comparison, the fat content was nearly five times lower than that of dietary fiber and four times lower than that of protein. Our previous research confirmed the low fat content (3.52–6.72 g/100 g dry weight (DW)), high levels of fiber (17.25–36.52 g/100 g DW), and available carbohydrates (16.55–46.15 g/100 g DW) in other cultivars of red radish microgreens [26]. Similarly, Lone et al. [29] reported fat as the least abundant macronutrient and protein as the predominant one in pink radish microgreens. However, other authors found that the soluble sugar content was six times higher than protein levels, whereas in our study, the difference was only 24% [22]. The total dietary fiber content of the radish microgreens was 28.37 g/100 g, with the insoluble fraction accounting for 90.91% of the total. Primary plant metabolites are accompanied by structurally very diverse secondary metabolites, including vitamins, carotenoids, chlorophylls, and phenolic compounds. The total contents of lipophilic pigments, total phenolics, proanthocyanidins, and vitamin C in red radish microgreens are also presented in Table 1. Among secondary metabolites, phenolic compounds (562.27 mg/100 g) and chlorophylls (468.87 mg/100 g) were the most abundant. On the other hand, vitamin C was present in the lowest amount (7.94 mg/100 g). Similar relationships have been reported in other studies of radish microgreens [22,24,30,31]. Within the group of photosynthetic pigments, the total chlorophyll content was nine times higher than the content of total carotenoids. In addition, chlorophyll *a* was the predominant form, present at more than twice the content of chlorophyll *b*. This trend is consistent with findings from other studies, which also observed a higher content of chlorophyll *a* than chlorophyll *b* and a general predominance of chlorophylls over carotenoids in radish microgreens [5,32]. Despite numerous studies reporting total phenolic and flavonoid content in radish microgreens, data on tannin content remain limited and inconsistent [22,23,24,30,33]. In our study, the total proanthocyanidin content in freeze-dried radish microgreens was determined to be 185.90 mg/100 g. Proanthocyanidins, also called condensed tannins, are oligomeric or polymeric forms of flavanols. They exhibit considerable structural diversity due to differences in polymerization degree, hydroxylation patterns, and the types and positions of interflavan bonds [34]. A higher total tannin content (274 mg/100 g DW) in radish microgreens was reported by Šola et al. [22], although in that study, tannins were determined indirectly-based on the difference between the Folin–Ciocalteu reaction product of total polyphenols and that of the tannin-free fraction obtained by removing insoluble complexes with specific macromolecules.

### 2.2. Bioaccessibility and Bioavailability of Phenolic Compounds from Radish Microgreens

Bioaccessibility is the proportion of a substance released from the food matrix during digestion that becomes potentially available for absorption. Bioavailability refers to the fraction of ingested nutrients or bioactive compounds that reach the systemic circulation and are ultimately utilized [21]. So far, several in vitro digestion models have been developed to simulate the conditions of the human digestive tract and investigate food digestion, including a static in vitro digestion model developed by the INFOGEST international consortium [20,35]. This relatively simple, rapid, and inexpensive model was also used in our research. An important step in the simulated digestion process is the separation of bioaccessible and bioavailable fractions. Filtration and centrifugation of digesta are used to separate insoluble undigested material from bioaccessible compounds. The bioavailable fraction can be separated using a dialysis method that mimics passive absorption of potentially bioavailable compounds, or by using cellular models (Caco-2, HT-29, IPEC-J2) [21]. In the present study, the bioaccessible fraction after gastric digestion was isolated by centrifugation, while the bioavailable fraction was isolated by dialysis during intestinal digestion. The effect of in vitro digestion of red radish microgreens on phenolic compound composition is shown in Table 2.

A total of twenty-two phenolic compounds were identified in undigested radish microgreens of the Warta cultivar, including one hydroxybenzoic acid, eight hydroxycinnamic acids, nine flavonols, and four anthocyanins. Flavonols were the most abundant class (305.64 mg/100 g), representing 54.41% of total phenolic compounds. The identified flavonols were mainly various kaempferol glycosides and acylated glycosides with *p*-coumaric or ferulic acids. The most representative flavonol in the undigested microgreens was kaempferol dirhamnoside (114.80 mg/100 g; 37.56% of total flavonols), followed by kaempferol 3-*O*-*p*-coumaroylglucoside (83.12 mg/100 g; 27.20% of total flavonols). Quantitatively, the second most abundant group of phenolic compounds in the analyzed microgreens was hydroxycinnamic acids (188.06 mg/100 g; 33.48% of total phenolics), with disinapoylgentiobiose as the dominant component (80.83 mg/100 g; 42.98% of total hydroxycinnamic acids). Anthocyanins were present in the smallest amounts (29.87 mg/100 g). The identified anthocyanins were nonacylated or acylated pelargonidin and cyanidin glycosides, with pelargonidin 3-rutinoside being the most abundant (17.88 mg/100 g; 59.86% of total anthocyanins). The results presented in Table 2 indicate a differentiated effect of in vitro simulated digestion on the phenolic compounds of radish microgreens, both with respect to the analyzed groups of phenolic compounds and the digestion phase. Compared to the undigested plant material, the bioaccessible fraction after gastric digestion contained statistically (*p* < 0.05) higher levels of total phenolics (an increase of 70.35%) and total hydroxycinnamic acids (a 3.5-fold increase). Statistically significant changes were also observed in the content of total flavonols (a decrease of 19.62%) and anthocyanins (a decrease of 42.58%). Such a significant increase in the level of hydroxycinnamic acids was associated with the appearance of feruloylmalic acid (380.80 mg/100 g) and *p*-coumaric acid (189.66 mg/100 g) in the gastric digesta. Moreover, the presence of protocatechuic acid hexoside, *p*-hydroxybenzoylhexoside, and sinapoylmalic acid was observed only in the bioaccessible gastric digesta. The changes in phenolic compound content observed in our study could probably result from the complete degradation of as many as five flavonol glycosides (including two acylated ones) and five hydroxycinnamic acids. Furthermore, two cyanidin derivatives and rhamnosyl-ellagic acid were completely decomposed under conditions simulating gastric digestion. On the other hand, the low pH and the action of pepsin in the stomach promote the softening of the food matrix and help release phenolic compounds. An increase in the content of total phenolics (by 40.26%) in the bioaccessible gastric fraction was also demonstrated by Šola et al. [22] for radish microgreens of the Cherry Belle cultivar. The authors also observed a similar effect for other Brassicaceae microgreens, such as kohlrabi, Savoy cabbage, Brussels sprouts, cauliflower, and garden cress [22]. The increase in phenolic compound content ranged from 15.39 to 36.42%, which was lower than that in our study. It should be noted that the total phenolic content in the cited work was determined by the spectrophotometric method with the Folin–Ciocalteu reagent, while our results refer to total phenolic content determined by ultra-performance liquid chromatography (UPLC). Furthermore, Šola et al. [22] observed significant changes in ferulic acid and kaempferol contents, while amounts of sinapic acid and quercetin were not significantly different.

Intestinal digestion of radish microgreens had a negative effect on the stability of phenolic compounds (Table 2). In relation to the undigested sample, a decrease in the concentration of total hydroxybenzoic acids (−53.30%), total flavonols (−73.09%), and total anthocyanins (−75.63%) was observed in the bioavailable fraction. In contrast, the total content of hydroxycinnamic acids increased 3-fold. However, comparing the gastric bioaccessible fraction to the intestinal bioavailable fraction, a decrease in the concentration of most compounds was observed, except for *p*-coumaric acid and caffeoylquinic acid hexoside. Tomas et al. [23] reported highly variable bioaccessibility of different phenolic compound groups in fresh purple radish microgreens, ranging from 2.9% for anthocyanins to 26.7% for lignans after gastrointestinal digestion. Bioaccessibility for total phenolics was 12.1%. Similarly, de la Fuente et al. [24] showed lower values of total phenolics and a lack of total monomeric anthocyanins in the accessible fraction after gastrointestinal digestion of freeze-dried radish microgreens. Bioaccessibility of total soluble polyphenols was 67.96%.

**Table 2:** Tentative identification and content (mg/100 g) of phenolic compounds before and after in vitro digestion of radish microgreens.

R_t_ (min)	λ_max_ (nm)	[M − H]^−^/[M + H]^+^ (*m*/*z*)	Tentative Identification	Undigested Microgreens	Gastric Bioaccessible Fraction	Small Intestine Bioavailable Fraction	Large intestine Bioavailable Fraction	Ref.
2.76–2.79	319	315	Protocatechuic acid hexoside	0.00 ^a^	28.66 ± 0.64 ^d^	17.83 ± 0.20 ^c^	8.87 ± 0.91 ^b^	[36]
3.22	279	447	Rhamnosyl-ellagic acid	38.18 ± 0.38 ^b^	0.00 ^a^	0.00 ^a^	0.00 ^a^	[37]
3.90	243	300	*p*-Hydroxybenzoyl hexoside	0.00 ^a^	8.67 ± 0.24 ^b^	0.00 ^a^	0.00 ^a^	[36]
Total hydroxybenzoic acids				38.18 ± 0.38 ^c^	37.33 ± 0.71 ^c^	17.83 ± 0.20 ^b^	8.87 ± 0.91 ^a^	
3.43	325	353	Caffeoylquinic acid	4.10 ± 0.17 ^b^	0.00 ^a^	0.00 ^a^	0.00 ^a^	[38]
3.49–3.56	315	547	Caffeoylsinapoyl hexoside	30.20 ± 0.49 ^c^	7.62 ± 0.86 ^b^	0.00 ^a^	0.00 ^a^	[39]
3.65	329	355	Feruloyl hexoside	43.14 ± 0.44 ^b^	0.00 ^a^	0.00 ^a^	0.00 ^a^	[40]
3.99	313	431	Sinapoyl hexoside	5.94 ± 0.81 ^b^	0.00 ^a^	0.00 ^a^	0.00 ^a^	[38]
4.63–4.65	314	163	*p*-Coumaric acid	0.00 ^a^	189.66 ± 5.97 ^c^	193.41 ± 9.82 ^c^	41.88 ± 3.36 ^b^	[40]
4.91–5.18	324	309	Feruloylmalic acid	0.00 ^a^	380.80 ± 8.58 ^c^	338.24 ± 30.11 ^c^	136.90 ± 10.03 ^b^	[41]
5.08–5.36	309	753	Disinapoylgentiobiose	80.83 ± 3.30 ^d^	68.39 ± 2.54 ^c^	18.45 ± 3.35 ^a^	26.38 ± 1.48 ^b^	[37]
5.39	309	723	Sinapoylferuloylgentiobiose	11.94 ± 1.96 ^b^	0.00 ^a^	0.00 ^a^	0.00 ^a^	[41]
6.45	312	537	*p*-Coumaroyl dihydromonotropein	4.50 ± 0.17 ^b^	0.00 ^a^	0.00 ^a^	0.00 ^a^	[42]
7.16–7.46	319	959	Trisinapoylgentiobiose	7.41 ± 0.07 ^c^	5.72 ± 0.16 ^b^	0.00 ^a^	0.00 ^a^	[41]
7.44	313	339	Sinapoylmalic acid	0.00 ^a^	4.61 ± 0.25 ^b^	0.00 ^a^	0.00 ^a^	[41]
9.02–9.33	312	515	Caffeoylquinic acid hexoside	0.00 ^a^	0.00 ^a^	7.10 ± 0.59 ^c^	4.67 ± 0.64 ^b^	[42]
Total hydroxycinnamic acids				188.06 ± 5.30 ^a^	656.80 ± 18.13 ^c^	557.20 ± 39.89 ^b^	209.83 ± 13.18 ^a^	
3.71–3.83	342	447	Kaempferol-7-glucoside	9.40 ± 0.07 ^b^	24.36 ± 1.62 ^c^	0.00 ^a^	0.00 ^a^	[41]
4.05–4.20	343	755	Kaempferol-3-*O*-glucosyl-rhamnosyl-glucoside	5.93 ± 0.10 ^c^	0.00 ^a^	0.00 ^a^	2.58 ± 0.17 ^b^	[43]
4.14	328	725	Kaempferol pentoside-rutinoside	6.44 ± 0.68 ^b^	0.00 ^a^	0.00 ^a^	0.00 ^a^	[44]
4.08–4.38	345	593	Kaempferol 3-*O*-*p*-coumaroyl glucoside	83.12 ± 1.85 ^c^	73.89 ± 4.17 ^b^	32.27 ± 2.94 ^a^	28.39 ± 1.04 ^a^	[45]
4.27–4.37	338	563	Kaempferol-3-*O*-arabinoside-7-*O*-rhamnoside	52.05 ± 0.47 ^d^	44.24 ± 1.08 ^c^	17.35 ± 1.57 ^b^	13.81 ± 1.24 ^a^	[45]
4.50–4.60	331	887	Kaempferol derivative	25.38 ± 0.39 ^c^	0.00 ^a^	0.00 ^a^	9.30 ± 0.29 ^b^	[45]
4.58–4.81	340	577	Kaempferol dirhamnoside	114.80 ± 1.79 ^d^	103.17 ± 2.01 ^c^	32.63 ± 3.03 ^a^	41.14 ± 0.52 ^b^	[44]
4.88–4.99	331	901	Kaempferol 3-*O*-(*p*-coumaroyl)dirhamnosyl hexoside	4.39 ± 0.38 ^b^	0.00 ^a^	0.00 ^a^	23.01 ± 1.84 ^c^	[46]
5.15	324	947	Kaempferol 3-*O*-feruloyldiglucoside-7-*O*-glucoside	4.13 ± 0.53 ^b^	0.00 ^a^	0.00 ^a^	0.00 ^a^	[47]
5.32	342	447	Kaempferol 7-*O*-glucoside	0.00 ^a^	0.00 ^a^	0.00 ^a^	20.29 ± 1.15 ^b^	[41]
5.65	343	417	Kaempferol 7-*O*-pentoside	0.00 ^a^	0.00 ^a^	0.00 ^a^	7.30 ± 0.30 ^b^	[44]
6.20	341	431	Kaempferol 3-*O*-rhamnoside	0.00 ^a^	0.00 ^a^	0.00 ^a^	20.58 ± 0.86 ^b^	[44]
Total flavonols				305.64 ± 5.36 ^d^	245.66 ± 4.23 ^c^	82.25 ± 7.40 ^a^	143.39 ± 4.70 ^b^	
4.3	507	595+	Cyanidin 3-(glucosyl)rhamnoside	2.21 ± 0.05 ^b^	0.00 ^a^	0.00 ^a^	0.00 ^a^	[48]
4.54	509	919	Cyanidin 3-(coumaroyl)sophoroside-5-glucoside	3.82 ± 0.03 ^b^	0.00 ^a^	0.00 ^a^	0.00 ^a^	[37]
4.58–4.91	507	579+	Pelargonidin 3-rutinoside	17.88 ± 0.08 ^d^	12.82 ± 0.30 ^c^	4.81 ± 0.36 ^b^	3.74 ± 0.59 ^a^	[48]
4.66–5.00	507	1019+	Pelargonidin-3-(feruloyl)diglucoside-5-(malonyl) glucoside	5.96 ± 0.08 ^d^	4.34 ± 0.12 ^c^	2.47 ± 0.10 ^b^	1.17 ± 0.11 ^a^	[48]
Total anthocyanins				29.87 ± 0.12 ^d^	17.15 ± 0.42 ^c^	7.28 ± 0.35 ^b^	4.91 ± 0.55 ^a^	
Total phenolic compounds				561.75 ± 7.13 ^b^	956.94 ± 29.52 ^d^	664.56 ± 46.96 ^c^	367.00 ± 19.77 ^a^	
Total proanthocyanidins				185.90 ± 5.04 ^c^	36.41 ± 1.01 ^b^	28.27 ± 0.19 ^a^	28.16 ± 1.33 ^a^	

Data correspond to the average ± standard deviation of three replicates, and different letter (a, b, c, d) superscripts within the same row indicate statistically significant differences at *p* < 0.05 between samples. The content of hydroxybenzoic acid derivatives was expressed as 4-hydroxybenzoic acid equivalents, hydroxycinnamic acid derivatives as 5-caffeoylquinic acid equivalents, kaempferol derivatives as kaempferol 3-glucoside equivalents, and anthocyanins as cyanidin 3-glucoside equivalents. Proanthocyanidins were determined after acid depolymerization and expressed as cyanidin equivalents.

For comparison, in our study, the bioavailability was estimated at 89.38% for total hydroxycinnamic acids, 61.98% for total phenolics, 54.44% for flavonols, 23.23% for hydroxybenzoic acids, and 16.44% for anthocyanins. Šola et al. [22] observed a significant decrease in ferulic acid, kaempferol, and total phenolic content, while sinapic acid and quercetin content remained unaffected by intestinal digestion.

The phenolic compounds available for absorption after ingestion can be influenced by interactions with other food matrix components or dietary ingredients. Phenolic compounds are capable of binding with proteins, starch, dietary fiber, and lipids via ester, ether, carbon–carbon, and hydrogen bonds [49,50,51,52]. Radish sprouts were reported to contain 971 mg/100 g DW of bound phenolic acids (gallic, caffeic, *p*-coumaric, ferulic, sinapic) and 5.14 mg/100 g of flavonoids (quercetin, kaempferol, luteolin) [53]. Lower levels of bound phenolic compounds were found in radish roots (252.46 mg/100 g DW), with vanillic acid as the dominant form, followed by coumaric, ferulic, and caffeic acids [54]. The increase in some phenolic compounds after gastric digestion could be caused by enzymatic degradation (especially of phenolic acids) associated with fiber or complex plant cell structures. Soluble dietary fiber can prolong gastric emptying time and thus delay phenolic compound absorption in the small intestine [17]. Regarding dietary lipids, some phenolic compounds with lipophilic properties (e.g., flavonol aglycones) may be micellarized with dietary fat, enhancing their bioavailability. Radish microgreens digested in this study contained 5.44 g/100 g of total fat and were not rich in this nutrient. However, the effects of dietary proteins on phenolic compound bioaccessibility are inconclusive, as studies suggest a decrease, an increase, or no effect. The content of total protein in radish microgreens was 21.05 g/100 g.

Phenolic compounds released from the food matrix but not adsorbed in the small intestine, along with those remaining in the matrix, pass to the large intestine, where they may be transformed by microbial activities. Microbial enzymes can further release phenolic compounds from the undigested matrix or modify them. In our study, commercial enzyme preparations (Pronase E and Viscozyme L) were used to mimic the action of microbiota occurring in the large intestine. Pronase E contains a mixture of bacterial proteases, and Viscozyme L includes a wide range of carbohydrolases (including cellulase, hemicellulose, arabanase, xylanase, and β-glucanase) [55]. Our results showed that the content of total phenolics, hydroxybenzoic acids, and anthocyanins decreased significantly in the large intestine bioavailable fraction compared to the undigested, gastric, and small intestine samples (Table 2). The total flavonol content in the large intestine fraction was lower than in the undigested microgreens and gastric bioaccessible fraction, but higher than in the small intestine bioavailable fraction. This increase in flavonol content was due to the presence of kaempferol derivatives (7-glucoside, 7-pentoside, and 3-rhamnoside), which were not detected in the initial sample or in earlier digestion phases. This may result from further release of matrix-bound flavonoids by the added proteases and carbohydrolases. It is worth underlining that this in vitro approach does not account for bacterial biotransformation. Human microbial enzymes can deconjugate phenolic compounds, allowing reabsorption of aglycones. In addition, intestinal microbiota can degrade aglycones into simpler aromatic compounds, such as hydroxyphenylpropionic acids and phenylvalerolactones (from flavanols), and hydroxyphenylacetic acids (from flavonols) [56].

Spectrophotometric analysis of radish microgreens also showed the presence of proanthocyanidins at a level of 185.90 mg/100 g (Table 1). However, no representatives of this group were identified by UPLC-MS (Table 2), which may indicate the presence of proanthocyanidins with a high degree of polymerization in the tested plant raw material, undetectable by this method. A higher polymerization degree results in more hydroxyl groups. The more hydroxyl groups there are, the more difficult the separation is. In the HPLC analysis, the peaks of each structural unit overlap and decrease, and the difficulty of structural identification increases as the polymerization degree increases [57]. Therefore, the total content of proanthocyanidins was determined spectrophotometrically after depolymerization to colored anthocyanidins using the acid–butanol test. Proanthocyanidin content decreased during subsequent digestion stages but did not differ statistically between the small and large intestinal bioavailable fractions. Compared to the gastric bioaccessible fraction, this reduction averaged 22.5%. Post-digestion levels were 5 to 6.6 times lower than in undigested microgreens, confirming the absence of low-polymerized, soluble proanthocyanidins and suggesting their binding to food matrix macromolecules, particularly fiber, as well as their low digestibility.

In vivo and in vitro studies on the proanthocyanidin metabolism were summarized by Niwano et al. [58]. Generally, oligomeric and polymeric proanthocyanidins reach the colon largely intact, where a small portion is degraded by microbiota to phenolic acids and valerolactones. Slight depolymerization may occur in the stomach and small intestine, but highly polymerized proanthocyanidins (>6 units) are remarkably stable in gastric environments. Their depolymerization has been observed in an in vitro small intestinal model using pancreatic enzymes and bile salts.

### 2.3. Effect of In Vitro Digestion of Radish Microgreens on Antioxidant Activity

Previous studies have shown that changes in the composition of radish microgreens, including phenolic compounds during the digestion process, affect their antioxidant activity [22,23,24]. In our study, the antioxidant properties of undigested radish microgreens, the bioaccessible gastric fraction, and the bioavailable small and large intestine fractions were determined by three methods: the potential to reduce ferric ion to ferrous ion (FRAP), scavenging activity toward the stable synthetic ABTS^•+^ cation radical (ABTS; 2,2′-azino-bis(3-ethylbenzothiazoline-6-sulfonic acid), and scavenging activity toward superoxide anion radical (SARSA). The determined antioxidant activity, expressed in Trolox equivalents (a water-soluble vitamin E analogue), is presented in Table 3.

According to all methods, the gastric bioaccessible fraction exhibited significantly higher antioxidant activity. The bioavailable fraction isolated at the stage of simulated digestion in the small and large intestine showed higher antioxidant activity compared to undigested microgreens. Moreover, the activities of these fractions did not differ significantly in a statistical sense. On the other hand, undigested microgreens were characterized by the lowest scavenging activity and reducing potential. The high antioxidant capacity of the gastric fraction may be due to the very high concentration of hydroxycinnamic acids and, consequently, the highest content of total phenolics. Conversely, attention should be drawn to the low antioxidant capacity of undigested microgreens, despite the highest levels of hydroxybenzoic acids, flavonols, anthocyanins, and proanthocyanidins. It should be emphasized that the antioxidant activity of undigested microgreens was determined in aqueous solutions obtained after extraction of the raw material with 80% ethanol and subsequent purification of the crude extract by solid-phase extraction. It is likely that not all proanthocyanidins, especially those with a high degree of polymerization, were isolated in this procedure. For comparison, Šola et al. [22] demonstrated a decrease in antioxidant activity of radish microgreens during gastric and intestinal digestion, measured by the ABTS and FRAP methods, while scavenging activity toward the DPPH radical was highest for the gastric digesta. However, gastrointestinal digestion of fresh radish microgreens resulted in a decrease in efficiency in scavenging synthetic ABTS^•+^ and DPPH^•^ radicals, as well as a decrease in reducing potential toward Fe^3+^ and Cu^2+^ ions (FRAP and CUPRAC test, respectively) [23]. A decrease in the antioxidant activity of freeze-dried radish microgreens subjected to simulated gastrointestinal digestion was also observed in other studies [24]. The bioaccessible fraction showed 54.27% and 28.18% of the initial activity in the ABTS and ORAC assays, respectively.

### 2.4. The Influence of In Vitro Digestion of Radish Microgreens on Biological Activity of Gut–Immune–Brain Axis Cells

**Figure 1:**
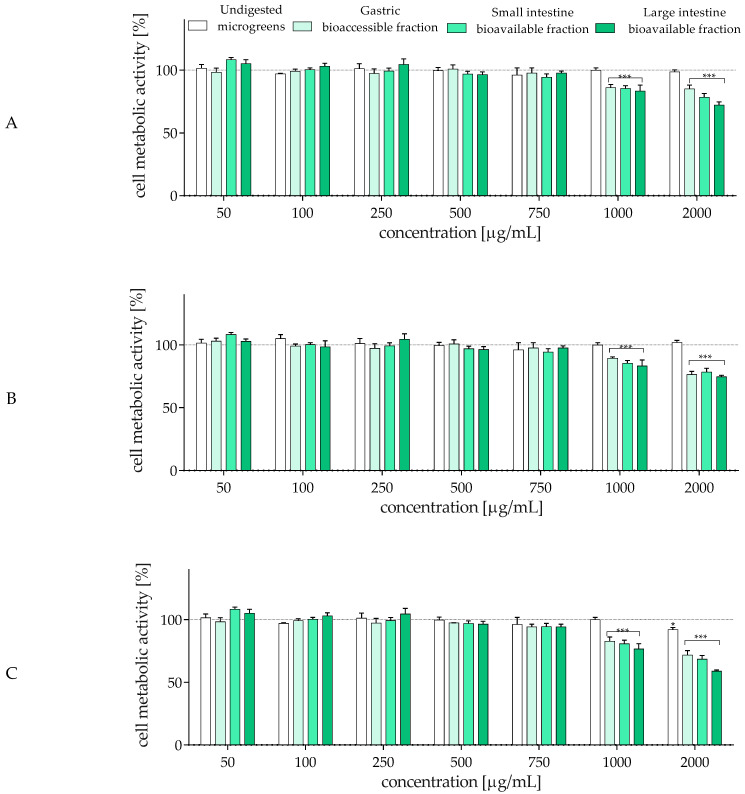
The effect of *R. sativus* microgreens (50–2000 µg/mL) on Caco-2 (**A**), HT-29 (**B**), and SH-SY5Y (**C**) cells’ metabolic activity, determined with the MTT assay after incubation for 24 h. The values in each column represent the mean value ± SEM, *n* ≥ 6. Control cells were only exposed to the vehicle (medium). Statistical significance was calculated against the control cells with * *p* ≤ 0.05, *** *p* ≤ 0.001.

In accordance with the Three Rs principles concerning ethical minimization of animal use in research (namely replacement, reduction, and refinement) [59], human-originated Caco-2 and HT-29 colon adenocarcinoma cell lines are most commonly used to reproduce a substitute for the epithelial lining [60]. In culture, these cells not only proliferate easily but also form tight junctions and differentiate, displaying structural and functional properties similar to intestinal enterocytes. Compared to Caco-2 cells, which mimic intestinal absorptive enterocytes, HT-29 cells exhibit relatively high expression and release of mucin. Whereas the co-culturing of Caco-2 and HT-29 cells is a model gaining popularity for investigating chemical absorption and bacterial adhesion, to obtain more precise data as a molecular basis for future detailed research, we decided to study the effect of the digestion process on the biological activity of radish microgreens on each type separately. First, the effect of microgreens on cell metabolic activity was assessed with the MTT test. All samples were studied within the range 50–2000 µg/mL, and as demonstrated in Figure 1A,B, undigested microgreens, even at the highest concentration, had no negative impact on cellular metabolism. On the other hand, all digested samples revealed cytotoxic potential, which at 1000 µg/mL decreased the metabolic activity of Caco-2 and HT-29 cells by 10–15% compared to the control. The cytotoxic activity observed at a concentration of 1000 µg/mL for all bioaccessible fractions of microgreens, despite the undigested, is connected with the chemical composition of the fractions. According to Table 1 and Table 2, after in vitro digestion, the deconjugation of phenolic compounds from the microgreens’ matrix occurs, leading to aglycon release. Aglycons possess higher bioactive potential, such as antioxidant/prooxidant and cytotoxic agents. These molecules are smaller and can be better absorbed or incorporated by cells or can affect cell membrane fluidity, thus impacting metabolic activity and signal transduction. At the highest studied concentration, samples from microgreens digested in the large intestine were the most cytotoxic, decreasing metabolic activity by 25–30%.

It should be emphasized that overall intestinal health relies not only on enterocytes’ homeostasis. Growing evidence directly links the gut with the nervous system and immune cells, as they are involved in the formation of the gut–immune–brain axis [61,62].

Considering that the gut contains the vagus nerve and the enteric nervous system, both identified as reward neurons providing important information to the brain [63], we determined the neuroprotective effect of the radish microgreen samples. The SH-SY5Y neuroblastoma cell line was used as a neuronal cell model, and Figure 1C demonstrates that these cells were more sensitive than epithelial-like cells. All digested samples decreased metabolic activity by 5–10% at 750 µg/mL, whereas incubation with 2000 µg/mL of the large intestine bioavailable fraction reduced SH-SY5Y metabolism to 55%. The study with neuron-like cells clearly demonstrated the effect of digestion on the bioactivity of cells. The cytotoxic pattern can be summarized by the increasing potential of microgreens: undigested < accessible gastric fraction < available small intestine fraction < available large intestine fraction. This pattern correlates with the quality and quantity of compounds released after each digestion step from their previously bound and conjugated forms; as small-molecule metabolites, they can more directly affect cellular metabolism and activity. Nevertheless, this research aimed to clarify the biological effect of microgreens using in vitro cell lines mimicking the gut–immune–brain axis. Therefore, for subsequent studies, the highest non-cytotoxic concentration observed for SH-SY5Y cells (IC_0_ = 500 µg/mL) was used, as it was lower than that observed for enterocytes (750 µg/mL).

**Figure 2:**
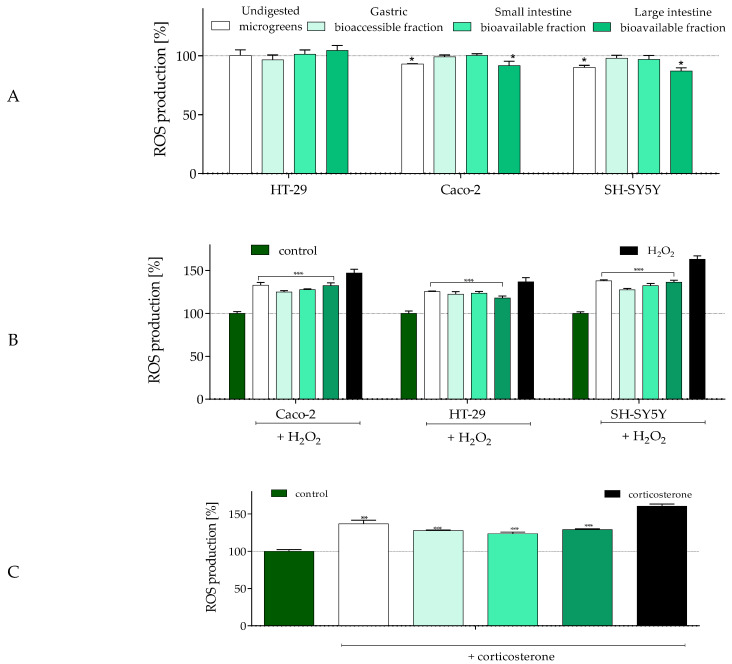
The effect of *R. sativus* microgreens (500 µg/mL) on SH-SY5Y cells’ intracellular ROS concentration, determined with the DCFH-DA assay (**A**); cytoprotective effect on oxidative stress induced with 250 µM H_2_O_2_ (**B**); cytoprotective effect against oxidative stress induced with 200 µM corticosterone (**C**). Values in each column represent the mean value ± SEM, *n* ≥ 6. Control cells were only exposed to the vehicle (medium). Statistical significance was calculated against the control cells in (**A**), whereas in (**B**,**C**), it was calculated against stressor-treated cells (H_2_O_2_ or corticosterone, respectively) with * *p* ≤ 0.05, ** *p* ≤ 0.01, and *** *p* ≤ 0.001.

Phytocompounds present in plant-originated food, such as phenolic compounds, can significantly affect the level of reactive oxygen species (ROS) generated in the digestive tract. Therefore, the effect of microgreens (IC_0_) on intracellular ROS level was studied (Figure 2).

As presented in Figure 2A, the most active in ROS reduction were undigested microgreens and the available small intestine fraction, which decreased intracellular ROS levels by 10–15% in Caco-2 and SH-SY5Y cells compared to the control. In the case of HT-29 cells, all samples had no impact on ROS levels—these cells secrete mucus that forms an additional barrier protecting cell membranes against direct interaction with compounds [60]. However, the lack of observed response may also result from the concentration being too low to be efficient for these cells. A study by de la Fuente et al. [8] confirmed the lack of cytotoxic effect of the bioaccessible fraction of radish microgreens on normal human colon fibroblasts CCD18-Co. However, simultaneously, this fraction enhanced ROS generation, decreased GSH levels, and impaired mitochondria in Caco-2 cells, which was followed by apoptosis induction. Here, the goal was to check the potential beneficial effect of microgreen fractions without the induction of any cytotoxic effect. On the other hand, the selected concentration agrees with Vučetić et al. [5], who did not observe cytotoxic effects of Sango radish extract on HCT116 (human colon carcinoma) and MRC-5 (non-tumor) cells at concentrations up to 1000 µg/mL.

Whereas reactive oxygen species (ROS) at low concentrations act as mediators playing a crucial role in the regulation of cellular responses, their excessive accumulation significantly affects the chemical structure of intracellular components, leading to protein and lipid oxidation and peroxidation, disruption of cell membranes, and DNA damage. One of the key detrimental factors affecting food and health is oxidative damage; hence, there is a growing interest in antioxidant preparations of natural and plant origin that can be incorporated into the everyday diet. Although antioxidative phytocompounds present in low doses offer health benefits, their excessive intake (either as food additives or supplements) may cause adverse effects, including the generation of oxidative stress. This, in turn, can disturb the gut microbiota and contribute to the development of chronic diseases, such as inflammatory bowel disease or colon cancer [62]. Microgreens, being easy and inexpensive to cultivate, are potentially important sources of dietary bioactive compounds. Therefore, their protective effect against oxidative damage in enterocytes and neurons is worth evaluating. One of the most commonly used chemical inducers of oxidative stress in in vitro cell line-based experiments is hydrogen peroxide (H_2_O_2_), which generates hydroxyl radicals via the Fenton reaction—considered among the most harmful ROS [64]. To assess the cytoprotective effect of microgreens, cells were incubated with H_2_O_2_ to induce oxidative damage, and the intracellular ROS level was determined. As demonstrated in Figure 2B, incubation with H_2_O_2_ increased oxidative stress levels by 35–65% across all cell types compared to the untreated control. HT-29 enterocytes were the most resistant, whereas neuronal cells exhibited the highest ROS elevation. Co-incubation with all radish samples showed a protective effect against induced ROS generation, reducing the cytotoxic impact of the oxidant by 15–30% in comparison to oxidant-treated cells alone. Interestingly, digested samples were up to 10% more effective in lowering ROS levels than undigested ones. The observed correlation between the digestion phase and antioxidant activity in Caco-2 and SH-SY5Y cells suggests that the gastric bioaccessible fraction had a stronger cytoprotective potential than undigested microgreens. In contrast, in HT-29 cells, the bioavailable small intestine fraction was the most effective. These findings are supported by the H_2_O_2_-induced lactate dehydrogenase (LDH) release assay, which reflects membrane damage (Figure 2B). Exposure of Caco-2 cells to the prooxidant increased LDH leakage to nearly 60% in comparison to untreated cells. Samples without digestion, as well as those digested in the small and large intestine, significantly reduced LDH release (by 30%). In HT-29 cells, all tested samples protected membrane integrity, with LDH levels in the culture medium comparable to those of control cells. These results are very promising, as preserving membrane integrity is crucial for maintaining the mucosal barrier [62]. Thus, it can be suspected that microgreens may protect the intestinal epithelial cell barrier against its disruption and dysfunction, particularly by preventing the infiltration of blood-derived inflammatory cells and gut-associated inflammation. However, this hypothesis requires further detailed studies using differentiated and co-cultured cell models.

Neuronal cells can also be damaged by corticosterone, an endocrine factor associated with gut dysbiosis, prolonged stress, and depression [65,66]. Elevated corticosterone concentrations increase intestinal and blood–brain barrier permeability, activate peripheral immune responses, and enhance oxidative stress in the central nervous system. Incubation of SH-SY5Y cells with corticosterone (Figure 2C) confirmed the neuroprotective properties of the microgreen samples. Treatment with hormone alone increased intracellular ROS levels by 60% in comparison to control cells, whereas co-incubation with radish microgreens reduced ROS levels by 20–35%. Among the tested variants, undigested microgreens exhibited the weakest protective effect, while the most efficient were the digested samples, especially those from the bioavailable small and large intestine fractions. These findings suggest that microgreens could serve not only as a source of nutrients but also as a reservoir of phytocompounds capable of modulating cognitive function or depression via the gut–brain axis. This is particularly relevant for addressing psychoneurological symptoms associated with cancer treatment [67].

**Figure 3:**
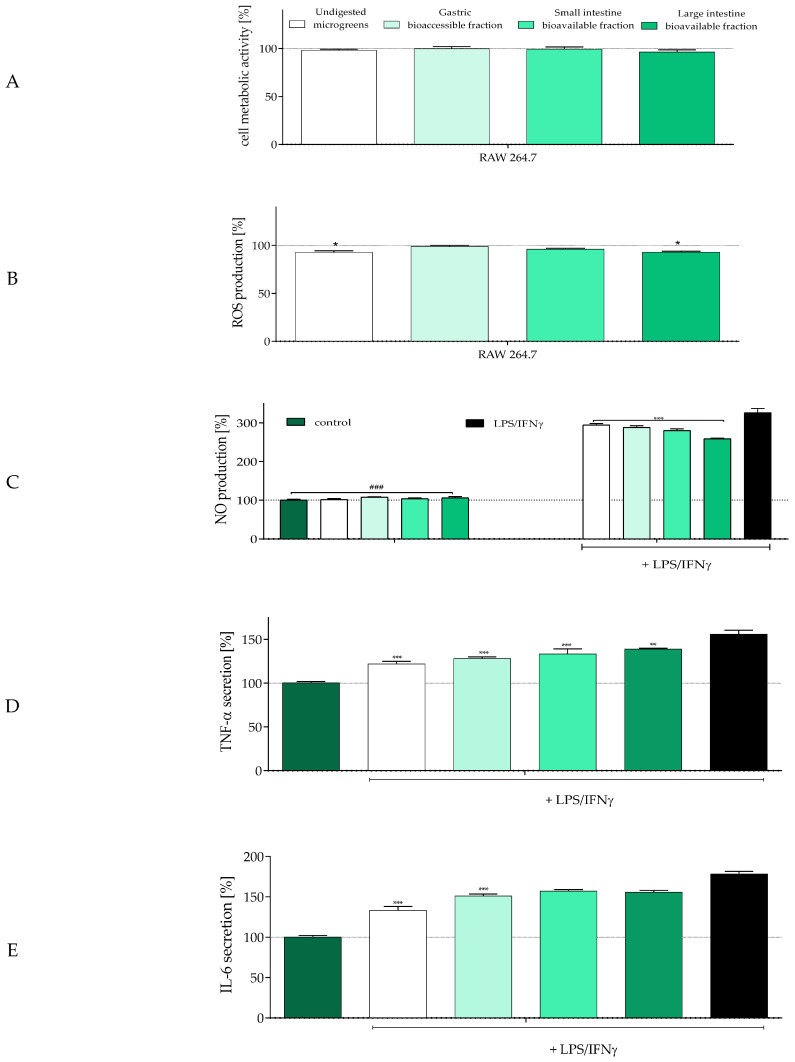
The effect of *Raphanus sativus* microgreens (500 µg/mL) on RAW 264.7 metabolic activity determined with the MTT assay after incubation for 24 h (**A**); intracellular ROS concentration determined with the DCFH-DA assay (**B**); the effect on nitric oxide (NO) production determined with the Griess reagent (**C**); secretion of interleukin-6 determined with ELISA (**D**); secretion of tumor necrosis factor α determined with ELISA (**E**). Control cells were only exposed to the vehicle. The values in each column represent the mean ± SEM, *n* ≥ 4. Significance differences in (**A**,**B**) were calculated against control cells, in (**D**,**E**) they were calculated against LPS/INF-γ-treated cells with ** *p* ≤ 0.01, and *** *p* ≤ 0.001, whereas in (**C**) against control cells with ^###^ *p* ≤ 0.001 and LPS/INF-*γ*-treated cells with * *p* ≤ 0.05, ** *p* ≤ 0.01, and *** *p* ≤ 0.001.

Today, there is no doubt that socio-environmental cues and dietary components are closely linked to the regulation of inflammation through the activation of neuro-inflammatory signal transduction and disruption of the intestinal barrier [62]. The presence of inflammatory mediators (such as TNF-α and IL-6) and oxidative stress strongly affects the gut–immune–brain axis, leading to epithelial barrier dysfunction, systemic inflammation, dysbiosis, and depressive disorders [68]. Therefore, in the next step, we investigated the modulatory effect of radish microgreens on the secretion of proinflammatory mediators by RAW 246.7 macrophages stimulated with lipopolysaccharide (LPS) and interferon γ (IFNγ) (Figure 3).

The samples, used at a previously established concentration equal to 500 µg/mL, had no statistically significant effect on macrophage metabolic activity (Figure 3A). However, two variants (undigested and the bioavailable small intestine fraction) decreased ROS levels by almost 10% in comparison to control cells (Figure 3B). Stimulation of macrophages with LPS and IFNγ led to the release of proinflammatory mediators related to diet-induced chronic diseases: nitric oxide (NO), interleukin-6 (IL-6), and tumor necrosis factor α (TNF-α). As shown in Figure 3C, treatment with LPS/IFNγ increased NO levels in the culture supernatant nearly 3-fold compared to unstimulated cells. All microgreen samples demonstrated an inhibitory effect on NO production, with the bioavailable small intestine fraction being the most effective, reducing NO levels by 60% compared to stimulated cells. These results suggested that the potential of *R. sativus* microgreens to inhibit NO increases along the digestive process. However, further studies demonstrated that this trend did not extend to TNF-α and IL-6 secretion. In both cases, the strongest suppression was achieved by undigested microgreens (Figure 3D,E), which reduced TNF-α and IL-6 release by 30% and 45%, respectively. However, digested samples still effectively inhibited cytokine production, although to a lesser extent (15–20% reduction compared to LPS/IFNγ-stimulated cells). Still, none of the studied radish microgreen samples stimulated NO production in non-stimulated macrophages, suggesting that radish microgreens lack immunostimulatory properties and rather exhibit anti-inflammatory potential by reducing the production of harmful mediators such as NO or H_2_O_2._ These mediators are known to negatively affect enterocytes and neurons, potentially contributing to intestinal barrier dysfunction, macrophage infiltration, uncontrolled immune responses to commensal intestinal microbiota, systemic inflammation, and mood disturbance [62].

A direct comparison of the results demonstrates that, in most cases, digested radish microgreens exhibited greater biological activity than undigested ones. This may be attributed to changes in phenolic composition throughout the digestion process. Each digestive phase alters the profile and quantity of released phenolic compounds, with increased levels of hydroxycinnamic acids and kaempferol glycosides. The observed reduction in total hydroxybenzoic acids, flavonols, and anthocyanins suggests that the biological activity depends more on the quality and potential synergism of the compounds than on their absolute quantity. Studies on phenolics from raspberry dried fruits or seeds have shown that the antioxidant capacity and α-glucosidase inhibition potential vary depending on the digestion stage, with gastric and small intestine digests being the most active [69]. Additionally, plant cultivation conditions may also influence radish microgreen chemical composition. For example, radish microgreens harvested in autumn are richer in caffeoylmalic acid, which has been associated with greater inhibition of lipogenesis in 3T3-L1 cells [13]. In another study, an extract of rat-tailed radish microgreen showed anticancer activity in HepG2 cells, with an IC50 of 612.5 ± 24.7 µg/mL. The treatment altered amino acid metabolism, including that of alanine, aspartate, glutamate, cysteine, and methionine. However, that study did not include a detailed chemical analysis of the microgreens [6]. Despite the lack of this information in some reports, radish microgreens, as members of the Crucifeorae family, are a rich source of phenolic compounds, isothiocyanates, and glucosinolates, which are known to have beneficial effects on human health through their anti-inflammatory and antioxidant potential [4]. The search for phytocompounds that effectively regulate the gut–immune–brain axis has already begun to attract significant scientific interest [67]. The phenolic compounds present in *R. sativus* microgreens exhibit a wide range of health-regulating effects and may play an important role in supporting the gut microbiota in protecting against dysbiosis and reducing the incidence of chronic diseases such as metabolic disorders, mental disorders, or even aging.

## 3. Materials and Methods

### 3.1. Standards and Reagents

Hyper grade formic acid for LC-MS was purchased from Carl Roth (Karlsruhe, Germany). Cyanidin 3-glucoside and 5-caffeoylquinic acid were obtained from Extrasynthese (Lyon, France). Kaempferol 3-glucoside was purchased from PhytoLab (Vestenbergsgreuth, Germany). All cell culture reagents were obtained from Life Technologies (Carlsbad, CA, USA). Boric acid, butanol, glacial acetic acid, hydrogen chloride, magnesium chloride, petroleum ether, potassium chloride, potassium phosphate monobasic, sodium carbonate, sodium carbonate monobasic, sodium chloride, and sodium hydroxide were purchased from Chempur (Piekary Śląskie, Poland). Calcium chloride, Folin–Ciocalteu reagent, and ethanol were obtained from POCH (Gliwice, Poland), and acetone, *n*-hexane, methanol, and sulphuric acid were obtained from POCH BASIC (Avantor Performance Materials Poland S.A., Gliwice, Poland). All other reagents were supplied by Sigma-Aldrich (Steinheim, Germany). Ultrapure water (Milli-Q Ultrapure Water SystemCorp., Bedford, MA, USA) was used to prepare all solutions.

### 3.2. Plant Material

Radish seeds of Warta cultivar, obtained from a commercial source (“W. Legutko” company, Jutrosin, Poland), were used as a material for microgreens cultivation. Microgreens were grown in 30 × 50 × 5 cm (7.5 L) microgreen trays placed on the tables in the growth chambers. The plants were grown on Hartmann’s universal substrate pH 5.5–6.5 ± 0.2 (in water) with the addition of PG Mix NPK 14:16:18 multi-nutrient fertiliser at 1.0–1.3 kg/m^3^ (±20%). After sowing, the seeds were covered with sand and generously sprinkled. During cultivation, the substrate was moistened every two days. The cultivation cycle ended when the microgreens developed their first true leaves (15 days after sowing). At harvest, the plants were cut with scissors just above the substrate. No pesticides or additional fertilisers were applied during cultivation. The plants were grown under controlled conditions in the growth chambers. During emergence, the temperature was 23 °C for the first three days, then 21/17 ± 2 °C (day/night). The relative humidity was 60–70%. The plants were cultivated under white LED light, and the photosynthetic photon flux density (PPFD) measured near the top of the plant was 230 ± 10 µmol m^−2^ s^−1^. The daily light dose was approximately 13.2 mol m^−2^ with a 16 h photoperiod.

Radish microgreens, after harvesting, were washed with tap water, followed by air drying. Then, the plants were frozen at −20 °C for 24 h and dried at −20 °C for 20 h under a vacuum pressure of 0.03 Mbar, and finally dried at 40 °C for 4 h (0.002 Mbar) (Martin Christ, Alpha 1–2/LD, Osterode am Harz, Germany). Freeze-dried microgreens were milled into fine powder in a domestic grinder and sealed in airtight glass vessels in a laboratory cabinet without light for further analysis.

### 3.3. Preparation of Crude Extract of Phenolic Compounds

The crushed radish microgreens (0.2 g) were suspended in 80% ethanol (10 mL) and extracted four times for 15 min at room temperature using ultrasonication. After each extraction step, the mixture was centrifuged at 5500 rpm for 10 min (Centrifuge MPW-351R, MPW MED. Instruments, Warszawa, Poland), and another portion of the extraction agent was added to the sediment. All supernatants were combined, 5 mL of deionized water was added, and the mixture was concentrated on a vacuum evaporator (T < 40 °C) to a volume of 5 mL to remove ethyl alcohol (Rotavapor R-3, Büchi, Flavil, Switzerland). The obtained aqueous extract of phenolic compounds was then purified on a Sep-Pak C18 cartridge.

### 3.4. Purification of Phenolic Compounds on Sep-Pak C18 Cartridges

The crude extract and fractions after digestion were purified on Sep-Pak C18 cartridges (10 g capacity, Waters Corp., Milford, MA, USA) according to the previously used procedure [70]. The phenolic compounds were eluted with methanol, and the eluate was evaporated under reduced pressure (T < 40 °C). The dry residues were dissolved in 2 mL of deionized water, and the obtained aqueous solutions were used in the analysis of the phenolic compounds profile and the determination of biological activity.

### 3.5. In Vitro Simulated Gastrointestinal Digestion

The in vitro digestion procedure of radish microgreens, including digestion in the oral cavity, stomach, and small intestine, was performed based on the INFOGEST protocol. Stock solutions of salivary fluid (SSF), gastric fluid (SGF), and intestinal fluid (SIF) were prepared according to Minekus et al. [35]. The following procedure was used:

*Oral phase*: 200 mg of freeze-dried radish microgreens was mixed with 3.0 mL of SSF, 0.375 mL of deionized water, and 0.125 mL of 0.03 M CaCl_2_(H_2_O)_2_. The samples were placed in a water bath for 3 min to equilibrate the mixture temperature to 37 °C, and then 7.5 mg of α-amylase was added. The total volume of the mixture was 3.5 mL. The mixture was incubated in a water bath with stirring (stirring speed 200, amplitude 4) at 37 °C for 2 min (Water bath shaker type 357, ELPIN-PLUS s.c., Lubawa, Poland).
*Gastric phase*: The oral bolus was supplemented with 1.88 mL of SGF, 12.5 µL of 0.03 M CaCl_2_(H_2_O)_2_ solution, and 0.164 mL of deionized water. After mixing, the pH of the mixture was adjusted to 3.0 with 1 M HCl, and 0.4 mL of pepsin solution prepared in SGF fluid (16 mg of pepsin per sample) was added. The total volume of the mixture was 6 mL. The samples were incubated in a water bath with stirring at 37 °C for 120 min. After gastric digestion, a portion of the samples was immediately acidified to pH 2 using 1 M HCl and centrifuged for 10 min at 5500 rpm to obtain a gastric bioaccessible fraction. Phenolic compounds present in this fraction were purified on a Sep-Pak C18 cartridge.
*Small intestinal phase*: Other portions of the gastric bolus were supplemented with 2.0 mL of SIF, 0.1 mL of 0.03 M CaCl_2_(H_2_O)_2_ solution, 0.35 mL of deionized water, and 0.5 mL of aqueous bile salt solution (0.25 g of bile salts per sample). After mixing, the pH of the mixture was adjusted to 7.0 with 1 M NaOH, and 40 mg of pancreatin per sample was added. The mixture was then transferred to the dialysis membrane, which was closed from the top and placed in a beaker containing 50 mL of PBS buffer at pH 7. The samples were incubated in a water bath with stirring at 37 °C for 120 min. After intestinal digestion, the OUT fraction with bioavailable compounds was acidified to pH 2 using 1 M HCl and purified on a Sep-Pak C18 cartridge. The residue inside the membrane (IN fraction) was then subjected to enzymatic hydrolysis using Pronase E and Viscozyme L.

### 3.6. Enzymatic Hydrolysis of Samples After In Vitro Digestion

The use of Pronase E and Viscozyme L for the hydrolysis of samples after simulated digestion was based on Castaldo et al. [71]. The intestinal digestion residue inside the dialysis tube was adjusted to pH 8, and 10 mg of Pronase E per sample was added. The membrane was closed at the top and placed in a beaker containing 50 mL of PBS buffer at pH 7. The samples were incubated in a stirred water bath at 37 °C for 60 min. Then, the buffer containing bioavailable compounds (fraction OUT) was acidified to pH 2 and stored in a refrigerator until the next day. The residue inside the membrane was adjusted to pH 4, and 43 µL of Viscozyme L was added. The membrane was closed at the top and placed in a beaker containing 70 mL of PBS buffer at pH 7. The samples were incubated in a stirred water bath at 37 °C for 16 h. The buffer containing bioavailable compounds (fraction OUT) was acidified to pH 2, combined with the sample obtained after Pronase E treatment, and purified on a Sep-Pak C18 cartridge according to the procedure described in Section 3.4.

### 3.7. Identification and Content of Individual Phenolic Compounds

UPLC-MS analysis was performed on an ultra-performance liquid chromatograph (Waters Acquity UPLC system, Milford, MA, USA) equipped with a binary pump, an autosampler, a column compartment, and a diode array detector. Briefly, samples were eluted with a gradient of solvent A (4.5% formic acid in ultrapure water) and B (acetonitrile) on an Acquity UPLC HSS T3 C18 column (150 × 2.1 mm, 1.8 µm; Waters) operating at 30 °C, as described in the previous work [72]. The runs were monitored at the following wavelengths: flavanols and hydroxybenzoic acids at 280 nm, hydroxycinnamic acids at 320 nm, flavonols at 360 nm, and anthocyanins at 520 nm. The gradient program was as follows: initial conditions 99% (A), 3 min 75% (A), 10 min 60% (A), 12.5 min 100% (B), and 15.0 min 99% (A). The flow rate was 0.45 mL/min, and the injection volume was 5 µL. The mass spectrometer was operating in the negative and positive mode for a mass range of 150–1500 Da, fixed source temperature at 100 °C, desolvation temperature 250 °C, desolvation gas flow of 600 L/h, cone voltage of 45 V, capillary voltage of 2.0 kV, and collision energy 50 V. Leucine enkephalin was used as a lock mass. The instrument was controlled by Mass-Lynx^TM^ V 4.1 software. The compounds were tentatively identified based on their UV–Vis spectra and MS and MS^2^ properties in comparison with the literature data. A quantitative analysis of identified phenolics was based on the standards as follows: 4-hydroxybenzoic acid for hydroxybenzoic acid derivatives, 5-caffeoylquinic acid for hydroxycinnamic acid derivatives, kaempferol 3-glucoside for kaempferol derivatives, and cyanidin 3-glucoside for cyanidin and pelargonidin derivatives. The results were expressed as mg per 100 g of freeze-dried microgreens.

### 3.8. Proximate Analysis

The elementary chemical composition of freeze-dried radish microgreens (dry matter, moisture, ash, protein, and fat contents) was determined using procedures described by Nollet [73]. Dry matter was estimated by drying at 105 °C to constant weight. Ash was determined through incineration of freeze-dried microgreens in a muffle furnace at 600 °C for 6 h, crude protein by the Kjeldahl method (N × 6.25) (Kjeldahl, Büchi India Pvt. Ltd.; Automated Distillation Apparatus, Medson S.c., Paczkowo, Poland), and crude fat by Soxhlet extraction with *n*-hexane. Determination of total dietary fiber and its soluble and insoluble fractions was described in the previous study [74]. Available carbohydrates were calculated by difference [100 − (moisture + fat + protein + ash + total dietary fiber)] [75]. The contents of all components were expressed as grams per 100 g of freeze-dried microgreens.

### 3.9. Extraction and Determination of Chlorophyll and Carotenoid Pigments

Ground freeze-dried radish microgreens (10 mg) with 1.5 mL of acetone (100%) were vortexed for 1 min and centrifuged at 10,000 rpm for 5 min. Another portion of acetone was added to the precipitate, and the process was repeated until the solvent was colorless. Absorbance of pooled acetone supernatants was measured at wavelengths 662 and 664 nm for chlorophyll *a* and *b* and 470 nm for carotenoids against acetone. The concentrations of chlorophyll *a*, chlorophyll b, and total carotenoids were calculated using the equation described by Costache et al. [76] and expressed as mg/100 g of freeze-dried microgreens.

### 3.10. Determination of Total Phenolics and Total Proanthocyanidins

Total phenolic content was determined using Folin–Ciocalteu reagent as described in previous work [72]. The absorbance of reaction mixtures was measured at 760 nm after 20 min incubation at ambient temperature, and the content of total phenolics was expressed as mg gallic acid equivalents per 100 g of freeze-dried microgreens. The total proanthocyanidins were determined after their acid depolymerization to the corresponding anthocyanidins and expressed as mg of cyanidin equivalents/100 g of freeze-dried microgreens as described by Rösch et al. [77].

### 3.11. Extraction and Analysis of Vitamin C

Freeze-dried radish microgreens (0.1 g) with 3 mL of 1% solution of metaphosphoric acid were vortexed for 5 min and centrifuged at 10,000 rpm for 10 min. The supernatant was filtered through syringe filters filled with regenerated cellulose (RC) with a pore size of 0.44 µm (Pureland, Chemland, Stargard, Poland). L-ascorbic acid in the supernatants was determined via the HPLC system with a photodiode array detector (Water Corp., Milford, MA, USA). Separation was achieved on an ion exclusion Rezex ROA-Organic H^+^ column (300 × 7.8 mm, Phenomenex, Torrance, CA, USA) according to the procedure described by Aubert et al. [78]. The elution system was 0.005 N sulfuric acid, running isocratically at a flow rate of 1 mL/min. The wavelength of the detector was set at 245 nm. The ascorbic acid content was quantified using a calibration curve and expressed as mg of L-ascorbic acid per 100 g of freeze-dried microgreens.

### 3.12. In Vitro Antioxidant Activity Assays

The ability of undigested and digested fractions obtained from radish microgreens to scavenge ABTS radical cation, superoxide anion radical (SARSA method), and to reduce ferric ion (FRAP method) was investigated by the procedure described in previous work [74]. The antioxidant activity in all use methods was expressed as Trolox equivalents per 1 g of freeze-dried microgreens.

### 3.13. Cell Culture and Treatment

All cell culture experiments were performed in a humidified 5% CO_2_ and 95% atmosphere at 37 °C. All the experimental measurements were performed using the Synergy 2 BioTek Microplate Reader (BioTek, Winooski, VT, USA). *Raphanus sativus* aqueous solutions (Section 3.4) were diluted with culture medium to obtain the working solution, which was used to prepare all tested concentrations. In this study, with selected cell lines resembling cells present in the digestive intestinal tract, the biological effect of preparations was observed after 24 h of incubation. In all experiments, biological effect was calculated as the percentage of the value obtained for cells incubated with compounds in comparison to control cells treated with the medium. The cytotoxic effect of components of the digestion mixture in the preliminary study was not observed, and for clear presentation of results, untreated control cells were used.

Human colorectal adenocarcinoma HT-29 and Caco-2, human neuronal SH-SY5Y, and mouse macrophage-like RAW 264.7 cells were obtained from American Type Culture Collection (ATCC, Manassas, VA, USA). HT-29 and Caco-2 cells were grown in DMEM, whereas SH-SY5Y in DMEM: Ham’s F-12 (1:1); both media were supplemented with a 10% fetal bovine serum (FBS). RAW 264.7 cells were maintained in DMEM medium supplemented with 10% bovine calf serum (BCS). Cells were cultured in the presence of 100 U/mL penicillin, 100 µg/mL streptomycin, and 25 µg/mL amphotericin B.

### 3.14. Metabolic Activity

Metabolic activity was evaluated using colorimetric measurements with MTT (3-(4,5-Dimethylthiazol-2-yl)-2,5-Diphenyltetrazolium Bromide) reagent. Cells were seeded into 96-well plates at a density of 1 × 10^4^ cells/well in complete medium and grown overnight, and then incubated in the presence of the studied samples for 24 h. After this, 25 µL of MTT (5 mg/mL) was added, and cells were incubated for 3 h at 37 °C. After this time, MTT was removed, 100 µL of DMSO was added, plates were shaken for 5 min, and absorbance was read in a microplate reader at 570 nm [79].

### 3.15. Oxidative Stress Parameters

The intracellular generation of reactive oxygen species was investigated using the dichloro-dihydro-fluorescein diacetate (DCFH-DA) chemical. After 24 h of incubation with preparations, cells were washed with phosphate-buffered saline and incubated with 10 µM DCFH-DA for 30 min, and the fluorescent signal at F485/530 nm was analyzed. To determine the cytoprotective effect, cells after preincubation with a non-cytotoxic concentration of preparations for 24 h, were treated respectively with 250 µM H_2_O_2_ [64] or 200 µM corticosterone for 6 h [66]. The effect on membrane integrity and damage was based on the lactate dehydrogenase release determined by luminescence measurement with the LDH-Glo Cytotoxicity Assay (Promega, Madison, WI, USA).

### 3.16. Nitric Oxide (NO) Production and Secretion of TNFα and IL6

The level of NO production was determined in RAW 264.7 cells. Briefly, after cells attachment, they were treated with lipopolysaccharide (LPS, O55: B5 from Escherichia coli) (Millipore Sigma, Darmstadt, Germany) at 1 µg/mL and 100 U/mL interferon γ (IFN-γ) for 24 h in the presence of preparations studied [68]. After the cells’ treatment, the medium was collected and the accumulation of NO metabolite in the cell culture supernatant was measured using Griess reagent (1% sulfanilamide and 0.1% naphthylethylenediamine dihydrochloride; Sigma Aldrich, Steinheim, Germany), where 50 µL of the supernatant was mixed with 50 µL of Griess reagent (40 mg/mL) in a 96-well plate. After incubation at room temperature and darkness for 10 min, the absorbance was measured at 540 nm. Cells treated with LPS/IFN-γ without samples were used as the positive control of the inflammatory response. Simultaneously, in the supernatants after RAW 264.7 cells treatment the level of interleukin 6 (IL-6) (Mouse IL6 ELISA kit, Biorbyt Ltd., Cambridge, UK) and tumor necrosis factor α (TNF-α) (Mouse TNFalpha ELISA kit, Biorbyt Ltd., Cambridge, UK) released was determined using ELISA kits, following the manufacturer’s instructions [79].

### 3.17. Statistical Analysis

Data analysis was conducted using the Statistica 12.5 software (StatSoft, Kraków, Poland), with all measurements in triplicate and results shown as mean values with standard deviations (SDs). Analysis of variance (one-way ANOVA) and Tukey’s post hoc test were used to evaluate differences among fractions after digestion, identifying statistically significant differences at *p* < 0.05. All calculations of cell culture were evaluated for significance using one-way ANOVA followed by Dunnett’s test with the GraphPad Prism 6.0 software (GraphPad Software, Inc., La Jolla, CA, USA). The results presented in graphs were compared with control cells exposed to the vehicle only, where the values in each column represent the mean ± SEM, *n* ≥ 4. Significance differences were calculated against control cells or cells incubated with the LPS/INFγor corticosterone or H_2_O_2_-treated cells with * *p* ≤ 0.05, ** *p* ≤ 0.01, *** *p* ≤ 0.001, as it is presented in the figures’ captures.

## 4. Conclusions

Radish microgreens have gained much popularity because they are important and beneficial to the human body. In this study, for the first time, the biological properties of bioavailable fractions of the small and large intestinal digesta were comprehensively analyzed. Moreover, the use of different cell lines (Caco-2, HT-29; RAW 264.7 and SH-SY5Y), resembling the cells that form the gastrointestinal tract, regulating intercellular signaling mechanisms by modulating the gut–immune–brain axis allowed the verification of the activity of microgreens in different models. The results demonstrated that radish microgreens can be a valuable component of the human diet in supporting the body’s defense against oxidative stress and inflammation. Bioactive radish microgreen compounds released during gastric digestion, as well as those present in the potentially bioavailable fractions of the small and large intestine, did not have cytotoxic effects on Caco-2, HT-29, and SH-SY5Y cells at concentrations up to 1 mg/mL. Furthermore, they showed a protective effect by reducing ROS induced by hydrogen peroxide or corticosterone, inhibiting nitric oxide production, and suppressing cytokine secretion in RAW 246.7 macrophage cells. The antioxidant activity of radish microgreens after the simulated in vitro digestion was also confirmed by various chemical tests, demonstrating their ability to scavenge ABTS^•+^ and superoxide anion radicals, as well as to reduce iron(III) ions. The observed differences in biological activity between undigested and digested radish microgreen samples likely stem from the release and transformations of phenolic compounds influenced by changes in pH and enzymatic conditions during the digestion process. Compared to undigested microgreens, all samples after in vitro digestion were characterized by a higher content of hydroxycinnamic acids, especially feruloylmalic acid, which was not detected in the original plant material. Further studies are warranted to explore their interaction with food matrix components to assess their role when incorporated into complex meals. Moreover, the demonstrated health-promoting activity of radish microgreens after in vitro digestion needs to be verified in in vivo models involving humans.

## Figures and Tables

**Table 1:** Chemical composition of freeze-dried radish microgreens.

Components	Content (g/100 g)	Components	Content (mg/100 g)
Moisture	3.81 ± 0.10	Chlorophyll a	324.30 ± 13.09
Total protein	21.05 ± 0.34	Chlorophyll b	144.58 ± 6.67
Crude fat	5.44 ± 0.32	Total chlorophylls	468.87 ± 17.19
Available carbohydrates	26.17 ± 1.96	Total carotenoids	51.95 ± 4.43
Ash	15.16 ± 0.07	Total phenolics	562.27 ± 17.35
Total dietary fiber	28.37 ± 1.57	Total proanthocyanidins	185.90 ± 5.04
Insoluble dietary fiber	25.79 ± 1.49	Vitamin C	7.94 ± 0.33

Data correspond to the average ± standard deviation of three replicates.

**Table 3:** Antioxidant activity of freeze-dried radish microgreens before and after in vitro digestion.

Sample	ABTS	FRAP	SARSA
Undigested microgreens	18.78 ± 0.30 ^a^	18.25 ± 0.66 ^a^	121.81± 6.75 ^a^
Gastric biaccessible fraction	51.42 ± 4.54 ^b^	41.68 ± 2.39 ^c^	194.82 ± 13.44 ^c^
Small intestine bioavailable fraction	21.80 ± 1.90 ^a^	25.70 ± 0.63 ^b^	156.67 ± 5.82 ^b^
Large intestine bioavailable fraction	20.62 ± 1.71 ^a^	23.38 ± 2.03 ^b^	142.84 ± 16.43 ^a b^

Data correspond to the average ± standard deviation of three replicates. The means in columns with different letters (a, b, c) differ statistically at *p* < 0.05. ABTS and FRAP are expressed as µmpl Trolox/g, SARSA as mmol Trolox/g.

## Data Availability

Data are contained within the article.
